# Prevalence of Vitamin D Deficiency and Associated Risk Factors in the US Population (2011-2012)

**DOI:** 10.7759/cureus.2741

**Published:** 2018-06-05

**Authors:** Naveen R Parva, Satish Tadepalli, Pratiksha Singh, Andrew Qian, Rajat Joshi, Hyndavi Kandala, Vinod K Nookala, Pramil Cheriyath

**Affiliations:** 1 Internal Medicine, PinnacleHealth; 2 Internal Medicine, Hackensack Meridian - Ocean Medical Center, Edison, USA; 3 Internal Medicine, Penn State Milton S. Hershey Medical Center

**Keywords:** cholecalciferol, vitamin d deficiency, ergocalciferol, nhanes, cdc

## Abstract

Introduction

1,25-dihydroxyvitamin D3 (cholecalciferol), the hormonally active form of vitamin D3, is a lipid-soluble compound that plays a significant role in clinical medicine due to its potent effects on calcium homeostasis and bone metabolism. Since foods containing natural vitamin D are rare, the primary source of the compound remains its nonenzymatic dermal synthesis through exposure to ultraviolet rays in sunlight. Although uncommon in most developed countries, recent literature has demonstrated that subclinical vitamin D deficiency can exist in certain populations and plays a role in downstream clinical consequences, including cardiovascular disease, cancer, diabetes, osteoporosis, and fractures. This study aims to identify the prevalence and change in the pattern of vitamin D deficiency in subpopulations throughout the United States to provide a foundation for further clinical studies correlating the clinical outcomes to vitamin deficiency.

Methods

Data analyzed in this study were collected through National Health and Nutrition Examination Survey (NHANES), specifically from a population of 4962 participants, age ≥20 years, who were hospitalized between 2011 and 2012. This cohort was stratified to divide the population into patients that were vitamin D sufficient (>50 nmol/L) versus patients who were vitamin D deficient (50 nmol/L). The risk factors were compared between the subpopulations in 2005-2006 and 2011-2012.

Conclusions

The prevalence of vitamin D deficiency is greater in certain clinical subpopulations, and the presence of associated characteristics should raise the index of suspicion for the practicing clinician with regard to conditions associated with vitamin D deficiency, such as osteoporosis and osteomalacia. Further research investigating the pathophysiology of hypovitaminosis D and its clinical consequences can help better understand and prevent the development of associated comorbidities.

## Introduction

Vitamin D, a lipid-soluble vitamin, plays an essential role in maintaining skeletal integrity and function, electrolyte reabsorption, and immune system regulation among other health benefits. Vitamin D exists in two primary variants, vitamin D2 and D3. The hormonally active form of vitamin D, 1,25-dihydroxyvitamin D3 (cholecalciferol), plays a significant role in clinical medicine mainly due to its potent effects on calcium homeostasis and bone metabolism.

The primary source of vitamin D3 is sunlight, which contains ultraviolet rays that synthesize cholecalciferol in the skin [1]. Alternative sources of vitamin D3 include animal products, such as fatty fish, while vitamin D2 (ergocalciferol) can be obtained mainly from dietary plant and fungal sources such as mushrooms. Consequently, major risk factors for vitamin D deficiency include inadequate sunlight exposure, inadequate dietary intake of vitamin D-containing foods, and malabsorption syndromes such as Crohn’s disease and celiac disease [2]. Although clinically apparent manifestations of vitamin D deficiency, such as rickets in children or periosteal bone pain in adults, are uncommon in most developed countries, recent literature suggests that subclinical, asymptomatic vitamin D deficiency still plays a notable role in contributing to several of the leading causes of death, including cardiovascular disease, cancer, and diabetes.

According to data collected between 2005 and 2006 by the National Health and Nutrition Examination Survey (NHANES), insufficient vitamin D levels were found in 41.6% of the 4495-individual sample size. Race was identified as a significant risk factor, with African-American adults having the highest prevalence rate of vitamin D deficiency (82.1%, 95% CI, 76.5%-86.5%) followed by Hispanic adults (62.9%; 95% CI, 53.2%-71.7%) [3]. Additional risk factors for vitamin D deficiency that were identified included obesity, lack of college education, and lack of daily milk consumption [3].

This study aims to employ the most recently published NHANES data collected in 2011-2012 to identify the prevalence of hypovitaminosis D in subpopulations throughout the United States. Additionally, another objective of this study is to analyze trends in how rates of vitamin D deficiency are changing in these subpopulations with identified risk factors from 2005-2006 to 2011-2012. Multiple subpopulations previously unassociated with the risk of hypovitaminosis D were identified using this newly released data, including participants less than 65 years of age and participants who were cancer-free.

## Materials and methods

Data of participants were collected from NHANES, a program designed to study and assess the health and nutritional status of adults and children in the United States. The data from this site is released biannually and publicly available to researchers after data cleaning, editing, documentation, and Disclosure Review Board (DRB) review. As the data is available to researchers and is free of identifiers, no Institutional Review Board (IRB) approval and informed consent were required for this study.

Data analyzed in this study were collected through NHANES, specifically from a population of 4962 participants, 20 years of age and over. NHANES is a cross-sectional survey designed to monitor the health and nutritional status of the civilian non-institutionalized U.S. population. Data are collected and analyzed from a group of approximately 5000 individuals through the administration of standardized interviews and physical examinations that include laboratory tests utilizing blood and urine specimens. In 2011-2012, NHANES used a complex, multistage probability design to sample a civilian population from all 50 states, resolutely oversampling certain subgroups (non-Hispanic Asians, Hispanics, non-Hispanic African-Americans, older adults, low-income whites/others) to increase the reliability and precision of data analysis for these subgroups. With these data, NHANES estimated trends in the prevalence, awareness, treatment, and control of select diseases in the U.S. population as a whole and in designated subgroups with selected diseases and risk factors.

Although there are differences in opinion regarding the optimal level of vitamin D in the body, most experts agree that levels less than 50 nmol/L are suboptimal for skeletal health [4]. Therefore, this value was selected to divide the population into patients who were vitamin D sufficient (>50 nmol/L) and vitamin D deficient (<50 nmol/L). The population was further stratified based on demographic information (age, gender, etc.), common health conditions (hypertension, hypercholesterolemia, rheumatoid arthritis, cancer, etc.), and lifestyle factors (milk consumption, exercise, etc.) to assess the relative prevalence of hypovitaminosis D in these subpopulations. The presence or absence of these variables in correlation with vitamin D levels was analyzed using logistic regression with a 95% confidence interval. P-values <0.05 were considered statistically significant.

A descriptive statistical analysis was performed using SAS version 9.4 for Windows (SAS Institute, Cary, NC, USA). The chi-square test, student t-test, and the Pearson correlation coefficient were used for the data analysis.

## Results

The data of participants were collected from NHANES, a program designed to study and assess the health and nutritional status of adults and children in the United States. The data from this site are released biannually and publicly available to researchers after data cleaning, editing, documentation, and Disclosure Review Board (DRB) review. As the data is available to researchers and is free of identifiers, no IRB approval and informed consent were required for this study.

Of the 4962 participants interviewed in this study by NHANES 2011-2012, 1981 (39.92%) were vitamin D deficient (serum levels less than 50 nmol/L), a proportion that has remained consistent since 2005-2006, when NHANES found 40% of the population to be vitamin D deficient (Table 1).

**Table 1:** Distribution of vitamin D ( Age ≥ 20)

Vitamin D level	Number of People	Percent of People	Mean	Standard Deviation	Minimum	Maximum
<50 nmol/L	1981	39.92%	34.54	10.02	5.21	49.98
≥ 50 nmol/L	2981	60.08%	76.73	22.69	50	373.67
Total Population	4962					

When comparing this vitamin D deficient population to the remaining 2981 participants with sufficient vitamin D levels (greater than or equal to 50 nmol/L), several statistically significant correlations were found, each of which will be discussed below (Table 2).

**Table 2:** Risk factors for vitamin D deficiency

		95% Confidence Interval		
Variables	Odds Ratio	Lower Limit	Upper Limit	p-value
Age ≥ 65 years	0.52	0.44	0.61	<.0001
Race: African-American	3	2.61	3.44	<.0001
Some college	0.87	0.77	0.99	0.0351
Poor health	1.44	1.23	1.69	<.0001
Current smoker	1.12	0.97	1.31	0.1308
Obesity (BMI ≥ 30)	1.31	1.15	1.49	<.0001
Diabetes	1.11	0.92	1.35	0.2837
Cancer	0.69	0.54	0.89	0.0033
High total cholesterol (>200 mg/dl)	0.83	0.72	0.96	0.01
Consume milk products daily	0.63	0.55	0.71	<.0001

 Race

As previous studies indicate, significant differences in vitamin D levels were seen in non-Hispanic African-Americans and Mexican-Americans as compared to other races. Non-Hispanic African-Americans composed a significant 39.3% of the vitamin D deficient population, compared to only 16.5% of the vitamin D sufficient population (p-value <0.0001). To a lesser extent, significant differences were also seen in Mexican-Americans (Table 3).

**Table 3:** Demographics and risk factors

Variables	Vitamin D Level <50 nmol/L		Vitamin D Level		p-value
			≥ 50 nmol/L		
Total Population	1981		2981		
Age >65 years	294	14.84%	774	25.96%	<0.0001
Gender (Female)	1013	51.14%	1494	50.12%	0.4823
Race					
Mexican-American	237	11.96%	258	8.65%	0.0001
Other Hispanic	196	9.89%	312	10.47%	0.5149
Non-Hispanic White	377	19.03%	1497	50.22%	<0.0001
Non-Hispanic African-American	778	39.27%	493	16.54%	<0.0001
Others	393	19.84%	439	14.73%	<0.0001
Education					
Didn't finish high school	478	24.13%	664	22.27%	0.1285
Finished high school	443	22.36%	594	19.93%	0.0387
Any college education	1058	53.41%	1723	57.80%	0.0023
Health status					
Good/excellent	1279	64.56%	2186	73.33%	<0.0001
Poor/fair	476	24.03%	508	17.04%	<0.0001
Smoking status					
Never	14	0.71%	40	1.34%	0.0347
Former	365	18.43%	771	25.86%	<0.0001
Current	445	22.46%	536	17.98%	0.0001
Body weight					<0.0001
Underweight	55	2.78%	70	2.35%	
Normal	502	25.34%	987	33.11%	
Overweight	582	29.38%	997	33.45%	
Obese	842	42.50%	927	31.10%	
Hypertension	680	34.33%	1085	36.40%	0.1356
Diabetes	280	14.13%	348	11.67%	0.0107
High total cholesterol (>200mg/dL)	479	24.18%	840	28.18%	0.0027
Low HDL cholesterol (HDL <40 mg/dL)	359	18.12%	513	17.21%	0.3525
Consume milk products daily	564	28.47%	1256	42.13%	<0.0001
Thyroid stimulating hormone within normal range (0.4 - 4 mu/L)	603	30.44%	908	30.46%	0.9878
Gout	69	3.48%	141	4.73%	0.0326
Cancer	112	5.65%	300	10.06%	<0.0001
Coronary heart disease	58	2.93%	114	3.82%	0.0909
Congestive heart failure	69	3.48%	99	3.32%	0.7572
Liver disease	76	3.84%	121	4.06%	0.6941
Depression	306	15.45%	413	13.85%	0.1186
Psoriasis	58	2.93%	87	2.92%	0.9848
Minutes on walking or bicycling for travel on a typical day - mean (SD), range	63.8 (78.4)	10 - 720	64.7 (73.9)	10 - 600	0.8272

The established physiologic mechanism by which non-Hispanic African-Americans and Mexican-Americans are predisposed to vitamin D deficiency is the increased melanin levels found in the skin. The increased melanin absorbs and scatters ultraviolet rays from sunlight, which results in the less efficient conversion of 7-dehydrocholesterol to previtamin D3, a precursor of vitamin D. Therefore, dark-skinned individuals with higher melanin content will experience slower vitamin D synthesis in comparison to light-skinned individuals. To further support this physiologic mechanism, data from the study shows that non-Hispanic white participants accounted for only 19.03% of the vitamin D deficient population while making up 50.22% of the vitamin D sufficient population (p-value < 0.0001). This is significant because it serves as evidence that not only is high melanin content in the skin a risk factor for vitamin D deficiency, but lower melanin content acts as a protective factor.

Age

Although NHANES only gathered data from participants at least 20 years old, age was still found to be a statistically significant factor in this study.

A total of 14.8% of people aged more than 65 years of age were vitamin D deficient. This data surprisingly shows an increased incidence of vitamin D deficiency among participants aged under 65 years of age, thus refuting a commonly accepted notion that increasing age is directly proportional to the risk of being vitamin D deficient.

Education

Another statistically significant factor found in this study was education, specifically high school and college education. Out of the vitamin D sufficient population, 57.80% were college-educated compared to 53.41% of the vitamin D deficient population (p-value=0.0023). This finding supports the notion that college-educated consumers have better health awareness in general than those who never attended college, resulting in statistically significantly lower rates of vitamin D deficiency.

Health status

The health status of the participants in the study was divided into binary categories of either “good/excellent” or “poor/fair.” Among those who were vitamin D deficient, 64.56% had good or excellent health status compared to 73.33% of those who were vitamin D sufficient (p-value <0.0001). Similarly, 24.03% of vitamin D deficient participants had poor or fair health status compared to 17.04% of vitamin-D sufficient patients (p-value <0.0001). This variable is more difficult to extrapolate due to the nebulous nature of how each patient is categorized under an overarching health status. Regardless, the data support a direct correlation between the participants’ overall health status and their vitamin D levels.

Smoking

In this study, participants were divided into three different smoking categories: never smoked, former smoker, and current smoker. Rates of vitamin D deficiency were statistically significant among the “former smoker” and “current smoker” subpopulations but not the “never smoked” subpopulation. Among the vitamin D deficient population, 18.43% were former smokers compared to 25.86% of the vitamin D sufficient population, thus making vitamin D deficiency less prevalent among former smokers (p-value < 0.0001). An opposite trend was noted with current smokers; among the vitamin D deficient population, 22.46% were current smokers, whereas among the vitamin D sufficient population, 17.98% were current smokers (p-value <0.0001).

Recent studies have shown that the correlation between smoking and hypovitaminosis D may be related to the ability of sinus mucosa to activate circulating vitamin D levels. A recent study by Mulligan and colleagues examined the effects of cigarette smoke extract on primary human sinonasal epithelial cells, which showed a decreased expression for the gene that activates vitamin D3 in smokers [5]. This ability of cigarette smoke to impair vitamin D3 activation is a novel mechanism by which smoking induces pro-inflammatory and carcinogenic effects.

Body weight

The study showed statistically significant differences in vitamin D deficiency rates with respect to weight, with 42.5% of vitamin D deficient participants falling under the category of obese (BMI ≥30) compared to 31.1% of vitamin D sufficient participants (p-value <0.0001). This relationship between obesity and vitamin D deficiency is a consistent association found in published literature, with body fat content being inversely correlated to serum 25-hydroxyvitamin D concentrations [6]. Other factors that may contribute to this relationship include decreased mobility among obese patients, thereby decreasing sun exposure, as well as increased rates of bariatric or gastric bypass procedures, which may result in malabsorption of fat-soluble vitamins such as vitamin D.

Interestingly, the trend is reversed among overweight (BMI between 25 and 29.9) patients in this study, with 29.38% of vitamin D deficient participants being overweight compared to 33.45% of vitamin D sufficient participants (p-value <0.0001). Patients who are overweight but not obese are less likely to be prone to reduced mobility, leading to increased sunlight exposure, and they are not eligible for bariatric procedures that may induce a malabsorptive state and, consequently, hypovitaminosis D. Another hypothesis for why overweight patients are, in fact, less likely to be vitamin D deficient is that their diet may consist of more vitamin D-containing animal products than their non-overweight counterparts.

Daily milk product consumption

Milk fortification with vitamin D began in the United States during the 1930s, largely as an effort to combat rickets, a major public health problem at the time [7]. Almost all milk available in the U.S. is now fortified with 100 IU/cup of vitamin D, consequently, making rickets a rare disease in the United States. In this study, participants were interviewed to identify those who consumed milk products on a daily basis. Of the vitamin D deficient subpopulation, 28.47% consumed milk products daily, whereas 42.13% of vitamin sufficient participants consumed milk products daily (p-value <0.0001). These data support the efficacy of vitamin D-fortified milk products as a method of prophylaxis against hypovitaminosis D in conjunction with daily sun exposure.

Cancer

Vitamin D has long been established as a protective factor for cancer, with multiple studies showing an increased risk of cancer among vitamin D deficient patients, including colon, breast, ovarian, and prostate cancer [8]. In this study, however, there is a statistically significant (p <0.0001) higher rate of cancer found among the vitamin D sufficient population (10.06%) compared to the vitamin D deficient population (5.65%).

Diabetes

The relationship between vitamin D deficiency and diabetes has long been explored, with growing evidence suggesting vitamin D deficiency is a contributing factor to the development of both type 1 and type 2 diabetes. Identified mechanisms for this relationship include the presence of vitamin D receptors on the B-islet cells in the pancreas responsible for secreting insulin, as well as clinical improvement in diabetic patients who receive vitamin D treatment through increased insulin secretion [9]. Lastly, the role of vitamin D in calcium homeostasis, specifically its ability to reabsorb calcium in the small intestine and kidney, has been shown to indirectly regulate insulin secretion [9].

The data collected in this study support these findings, with 14.13% of the vitamin D deficient participants having diabetes compared to 11.67% of the vitamin D sufficient participants (p-value 0.0107). Other factors may contribute to this correlation between vitamin D deficiency and diabetes, including increased rates of obesity, kidney damage, and renal failure in diabetics, leading to impaired hydroxylation of 25-hydroxyvitamin D and, eventually, secondary hyperparathyroidism [10].

Non-significant results

The study stratified vitamin D deficient and sufficient populations by several other factors that yielded non-significant results, including gender, hypertension, thyroid-stimulating hormone (TSH), gout, coronary heart disease, congestive heart failure, liver disease, depression, and psoriasis. Additionally, daily minutes spent on walking or bicycling was another variable that proved to be non-significant, with similar results in both vitamin D deficient and sufficient subpopulations (p-value 0.83). 

## Discussion

Growing scientific evidence has implicated vitamin D deficiency in a multitude of chronic conditions, including type I diabetes, rheumatoid arthritis, hypertension, cardiovascular disease, and several common deadly cancers, among others [11]. With the growing prevalence of vitamin D deficiency across the United States and its association with these leading causes of mortality, it has become more important than ever to delineate vitamin D’s role in the pathogenesis of these diseases and use data to pinpoint established risk factors for vitamin D deficiency.

This study was conducted primarily to analyze the prevalence of vitamin D deficiency in the United States based on the latest published data collected by NHANES 2011-2012. Of the 4962 participants surveyed and examined, 1981 (39.92%) were found to be vitamin D deficient, which was in concordance with prior data collected in 2005-2006 (also by NHANES). A cross-sectional study was then conducted to further stratify the population to find factors associated with the risk of vitamin D deficiency, of which several were identified.

Primarily, this study supported established relationships between hypovitaminosis D and race, education, health status, smoking status, body weight, consumption of milk products, and diabetes. More specifically, the data from NHANES shows that there are statistically significant relationships between vitamin D deficiency and the non-Hispanic African-American as well as Mexican-American races, which was expected due to the increased levels of skin melanin found in these races on average. Similarly, data from this study show that there is an increased likelihood of vitamin D deficiency found among participants with diabetes, obesity, poor/fair health status, no college education, and current smokers. Lastly, the study supports the notion that the daily consumption of milk products serves as an effective prophylaxis against vitamin D deficiency.

Perhaps more interestingly, some of the data analyzed in this study refuted widely accepted associations between hypovitaminosis D and old age, as well as cancer. Prior studies have shown that increasing age is commonly associated with an increased risk of vitamin D deficiency, attributing this relationship to two main factors: the decreased efficacy of the human body’s synthesis of vitamin D through UVB-irradiation with age and the loss of mobility or residential care in the elderly that commonly restricts solar exposure.

Surprisingly, findings from this study deviate from the established notions that age correlates with the risk of vitamin D deficiency since we discovered that more participants under the age of 65 years of age were vitamin D deficient. Various confounding factors could contribute to these findings, but the surprising prevalence of vitamin D deficiency among individuals younger than 65 years of age compared to those older than 65 years is too drastic to be ignored. One primary factor that may play a role in decreasing the rates of hypovitaminosis D in older adults is the use of vitamin D supplementation, which has increased over time in the U.S. adult population. In one study by Rooney et al., less than 0.3% of the population had a daily supplemental intake of 1000 IU or more of vitamin D in 1999-2000, compared to 18.2% in 2013-2014 [12]. Today, over children and adolescents, on the other hand, the percentage of participants who took vitamin D supplements has remained relatively stable throughout the years [13].

Another proposed hypothesis for why vitamin D levels seem to be decreasing in the younger population is the exponential increase in technology such as big-screen television, computers, and gaming devices, making them inclined to stay indoors as compared to prior generations and, consequently, having less sunlight exposure. Additionally, because the average age of retirement in the United States is around 65 years, participants under this cutoff age may be spending most of their days working indoors during the time the sun is out. A 2001 study conducted by the National Human Activity Pattern Survey (NHAPS) to assess for exposure to environmental pollutants showed that over 90% of survey respondents spent time inside a residence from about 11 pm to 5 am, with respondents in schools, public buildings, offices, and factories spending time inside a residence between 7 am and 5 pm [14].

Our study showed that there was a statistically significant higher rate of cancer among the vitamin D sufficient population compared to those who were vitamin D deficient, thus contradicting strong evidence that vitamin D reduces the incidence and death rates of breast, prostate, colon, and ovarian cancers [8]. One factor that may contribute to this seemingly contradictory relationship may be the administration of vitamin D and other dietary supplements to patients diagnosed with cancer by their healthcare providers, whereas the vitamin D deficient population may be at high risk for cancer or even unaware of existing cancers and, consequently, not treated yet. Additionally, studies have also shown evidence that like low vitamin D levels, high vitamin D levels (>80 nmol/L) may also contribute to the increased rates of cancer through the development of vitamin D resistance [8].

## Conclusions

Although more research needs to be conducted to pinpoint the effects of age, occupation, technology, and cancer on vitamin D deficiency rates, the findings from this study are significant, showing that vitamin D deficiency is becoming an epidemic across the United States, even among groups that were not previously labeled “at-risk.” Healthcare professionals must be aware of the growing prevalence of vitamin D deficiency not only among patients with established risk factors, such as African-American or Mexican-American race, obesity, or diabetes but also among younger individuals and those who have no history of cancer.
